# Diagnosis and treatment of T/myeloid mixed phenotype acute leukaemia (T/M‐MPAL)

**DOI:** 10.1002/jha2.1075

**Published:** 2025-01-10

**Authors:** Ke Xu, Enas Abusalim, Evan Vitsaras, Karen Orfinada, Robert Baker, Elisabeth Nacheva, Andrew Wilson, Jenny O'Nions, Rajeev Gupta

**Affiliations:** ^1^ Department of Haematology University College London Hospital NHS Foundation Trust University College London London UK; ^2^ Specialist Integrated Haematology Malignancy Diagnostic Service Health Services Laboratories University College London Hospitals NHS Foundation Trust University College London London UK; ^3^ UCL School of Life and Medical Sciences London UK

1

Dear Editor,

T/myeloid mixed phenotype acute leukaemia (T/M‐MPAL) is a rare leukaemia subtype, probably accounting for <1% of all leukaemia cases [1]. It is characterised by immunophenotypic features of both myeloid and T‐lymphoid lineages. T/myeloid MPAL is distinct from T‐cell acute lymphocytic leukaemia (T‐ALL) and acute myeloid leukaemia (AML) but shares significant molecular and genomic similarity to early T‐cell precursor‐like ALL (ETP‐ALL). T/myeloid MPAL has a poorer prognosis than AML, T‐ALL and ETP‐ALL. Therefore, it is essential to make the correct classification. The study aimed to evaluate the T/myeloid MPAL diagnosis and review patients' treatment regimens and outcomes.

A retrospective analysis was performed of all T/M‐MPAL patients treated at University College London Hospital between February/2015 and April/2022 [2]. The data cutoff date was 29/September/2024. The diagnosis of T/M‐MPAL was made in accordance with the WHO diagnostic criteria [1]. Response assessments were made per European LeukemiaNet (ELN) criteria [3]. We reviewed bone marrow immunophenotyping (Beckman Coulter Duraclone), myeloid next‐generation sequencing (NGS) (Archer VariantPlex, and TruSight Illumina) (Tables S1 and S2), fluorescence in situ hybridization (FISH) analysis, and molecular karyotyping (8 × 60K oligonucleotide arrays, Agilent) results.  For flow cytometry, bone marrow samples were prepared using T‐Q Prep (Beckman Coulter), stained with a Duraclone kit (Beckman Coulter) (Table S3), and analysed on Navios flow cytometer (Beckman Coulter). Results were analysed using Kaluzo software (Beckman Coulter). Our standard diagnostic T/myeloid MPAL FISH panel consists of break apart or fusion probes targeting *KMT2A, CBFB::MYH11*, *RUNX1T1::RUNX1*, *PML::RARA*, *MECOM, TCRA/D* and probes targeting 5q, 7q, 20q and 17p (Cytocell).

Nine T/M‐MPAL cases were identified among the cases of leukaemia with a median follow‐up of 25 months [range 1–79 months] (Table 1). Of the nine patients, seven (78%) were male and two (22%) were female. The median age at diagnosis was 23 years old [range 13–73 years]. All patients' blast populations were positive for cCD3 (or CD3), MPO, CD34 and cCD34 and were negative for CD19 by flow cytometry. Myeloid markers CD117, CD13, and CD33 were positive at 66%, 88%, and 88%, respectively. T lymphoid markers CD2, CD5, and CD7 were positive at 55%, 44% and 100%, respectively (Table 1). Eight patients had NGS. The most common molecular abnormalities detected were *WT1* (62%), *NRAS/KRAS* (37%), and *BCOR* (25%). Additional mutations detected were *NOTCH* (12%), *RUNX1* (12%), *TP53* (12%), *IKZF1* (12%), *IDH2* (12%), and *U2AF1* (12%). Polymerase chain reaction (PCR) for *FLT3* ITD, *FLT3* TKD and *NPM1* was performed on all nine patients' samples. *FLT3* TKD was expressed at 11%, and *FLT3* ITD at 22%. Myeloid NGS did not detect *FLT3*‐ITD in patients 8 and 9 due to its lower sensitivity. None of the nine patients had *NPM1* mutation. All nine patients had FISH. Molecular karyotyping was performed on six patients' samples. Three patients' samples had G‐banding. One sample had complex karyotype with *ETV6* rearrangement; one showed T cell receptor (TCR) rearrangement; one showed *KMT2A* amplification and 17p deletion; one had trisomy 4 with a gain of *D4Z1* and one had deletion *TP53* with a gain of 7q; the remaining four had a normal karyotyping (Table 1).

**TABLE 1:** Patient's characteristics, treatment received and clinical outcome.

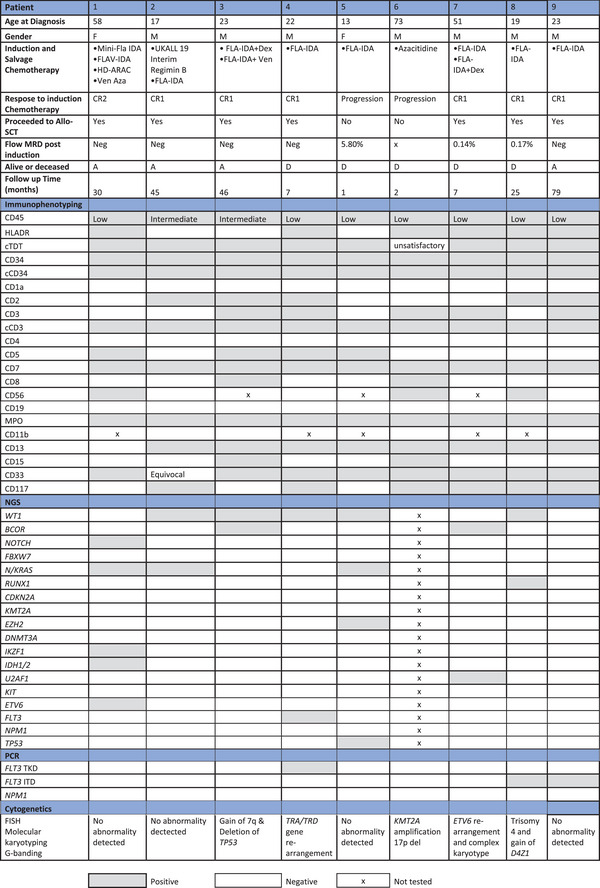

Abbreviations: *BCOR*, BCL6 corepressor; *CDKN2A*, cyclin‐dependent kinase inhibitor 2A; CR1, complete remission 1; CR2, complete remission 2; DA, daunorubicin and cytarabine; DEX, dexamethasone; *DNMT3A*, DNA (cytosine‐5)‐methyltransferase 3A; *EZH2*, enhancer of zeste homolog 2; *FBXW7*, F‐box/WD repeat‐containing protein 7; FLA‐IDA, Fludarabine, Cytarabine, Idarubicin; FLAV‐IDA, Fludarabine, Venetoclax, Cytarabine, Idarubicin; *FLT3*, Fms like tyrosine kinase 3; HD‐ARAC, high dose cytarabine; *IDH1/2*, isocitrate dehydrogenase 1 and 2; ITD, internal tandem duplication; *IKZF1*, the Ikaros zinc finger 1; *KMT2A*, lysine(K)‐specific methyltransferase 2A; *KRAS*, Kirsten rat sarcoma virus; Mini‐FLA‐IDA, dose reduced FLA‐IDA; *NRAS*, neuroblastoma rat sarcoma viral oncogene homolog; *NPM1*, nucleophosmin 1; PCR, polymerase chain reaction; *RUNX1*, Runt‐related transcription factor 1; TKD, tyrosine kinase domain; *U2AF1*, U2 small nuclear RNA auxiliary factor 1; Ven‐Aza, Venetoclax and Azacitidine; *WT1*, Wilms tumour protein 1.

One patient was unfit for intensive chemotherapy and received azacitidine with a palliative aim. Eight patients received FLA‐IDA (fludarabine, cytarabine, idarubicin), FLAV‐IDA (FLA‐IDA and venetoclax), or mini‐FLA‐IDA as part of an induction regimen. One patient required an alternative salvage regimen with venetoclax‐azacitidine before achieving remission. Seven patients achieved morphological complete remission (CR). Six of the seven CR patients also achieved flow cytometrical minimal residual disease (MRD) negativity post‐induction. All seven CR patients proceeded to allogeneic haematopoietic stem cell transplantation (alloHSCT) and remained in CR at the last follow‐up. Two patients did not proceed to alloHSCT due to disease progression. At the last follow‐up, four patients were still alive.

In our cohort of T/myeloid MPAL patients, the median age of diagnosis was 23 years, and 78% were male. Fifty‐six per cent of patients had cytogenetic abnormalities. Genetic mutations were identified in *WT1*, *NOTCH1*, *RAS*, *FLT3* ITD, *FLT3* TKD, *RUNX1*, *TP53, IKZF1, BCOR*, *ETV6*, *IDH2* and *U2AF1. WT1* was the most common mutation. The findings are in consistent with previous publications [4, 5, 6]. FLA‐IDA is effective in bridging to alloHSCT. The median OS was 25 months, and the 2‐year OS was 56%.

T/myeloid MPAL is a rare leukaemia subtype with a poor prognosis, and clinical management is challenging [4]. ETP‐ALL was defined on the basis of the following immunophenotypes: CD1a− (< 5% blast population), CD8− (< 5% blast population), CD5− or dim (< 75% of blasts population) and positivity for one or more stem cell or myeloid antigens [1]. A proportion of T/myeloid MPAL cases have immunophenotypic features that overlap with those of ETP‐ALL, and the only difference is MPO positivity in T/myeloid MPAL and MPO negativity in ETP‐ALL [1, 7]. In our centre, T/ myeloid MPAL patients receive a more intensified induction regimen, FLAG‐Ida, while the treatment for ETP‐ALL is an ALL‐directed induction regimen. Therefore, it is essential to make the correct diagnosis. A threshold of MPO of ≥3% was used to define positive MPO by cytochemistry [1], but currently, there is a lack of consensus on the cutoff of MPO positivity by flow cytometry. Flow cytometry thresholds for positive MPO vary between groups, ranging from 3%–20% [7, 8, 9, 10]. However, percentage cutoff points are unable to take into account the intensity of expression relative to normal counterparts [7]. Extra care should be taken to discriminate small MPO populations from background nonneoplastic myeloid progenitors. At our centre, laboratory haematologists, haematopathologists, leukaemia consultants, and flow cytometry scientists review all new acute leukaemia cases in multidisciplinary meetings to ensure the correct diagnosis. Future studies are needed to further standardise T/myeloid MPAL diagnosis.

## AUTHOR CONTRIBUTIONS

Ke Xu designed the study. Ke Xu and Enas Abusalim analysed the data and wrote up the manuscript. All the authors critically revised the manuscript.

## CONFLICT OF INTEREST STATEMENT

The authors have no conflicts of interest.

## FUNDING INFORMATION

The author(s) received no financial support for the research, authorship, and publication of this article.

## ETHICS STATEMENT

The authors have confirmed ethical approval statement is not needed for this submission.

## PATIENT CONSENT STATEMENT

The authors have confirmed patient consent statement is not needed for this submission.

## Supporting information



Supporting Information

## Data Availability

The datasets generated during and/or analysed during the current study are available from the corresponding author on request.
