# Supplementary material for: Diagnosis and treatment of T/myeloid mixed phenotype acute leukaemia (T/M‐MPAL)
Source: EJHaem. 2025 Jan 10;6(1):e1075. doi: 10.1002/jha2.1075 (PMC11756967; doi:10.1002/jha2.1075)
Supplement: Supplementary file 1 — Supporting Information [file JHA2-6-e1075-s001.docx]

**Supplementary Table 1**. List of genes on Archer VariantPlex myeloid NGS panel

| 75 Genes on Archer VariantPlex myeloid NGS panel | | | | |
| --- | --- | --- | --- | --- |
| *ABL1* | *CEBPA* | *GNAS* | *MYD88* | *SF3B1* |
| *ANKRD26* | *CSF3R* | *HRAS* | *NF1* | *SH2B3* |
| *ASXL1* | *CUX1* | *IDH1* | *NOTCH1* | *SLC29A1* |
| *ATRX* | *CXCR4* | *IDH2* | *NPM1* | *SMC1A* |
| *BCOR* | *DCK* | *IKZF1* | *NRAS* | *SMC3* |
| *BCORL1* | *DDX41* | *JAK2* | *PDGFRA* | *SRSF2* |
| *BRAF* | *DHX15* | *JAK3* | *PHF6* | *STAG2* |
| *BTK* | *DNMT3A* | *KDM6A* | *PPM1D* | *STAT3* |
| *CALR* | *ETNK1* | *KIT* | *PTEN* | *TET2* |
| *CBL* | *ETV6* | *KMT2A* | *PTPN11* | *TP53* |
| *CBLB* | *EZH2* | *KRAS* | *RAD21* | *U2AF1* |
| *CBLC* | *FBXW7* | *LUC7L2* | *RBBP6* | *U2AF2* |
| *CCND2* | *FLT3* | *MAP2K1* | *RPS14* | *WT1* |
| *CDC25C* | *GATA1* | *MPL* | *RUNX1* | *XPO1* |
| *CDKN2A* | *GATA2* | *MYC* | *SETBP1* | *ZRSR2* |

**Supplementary Table 2**. List of genes on TruSight Illumina myeloid NGS panel

| 54 Genes on TruSight Illumina Myeloid NGS Panel | | | | |
| --- | --- | --- | --- | --- |
| *ABL1* | *CEBPA* | *HRAS* | *MYD88* | *SF3B1* |
| *ASXL1* | *CSF3R* | *IDH1* | *NOTCH1* | *SMC1A* |
| *ATRX* | *CUX1* | *IDH2* | *NPM1* | *SMC3* |
| *BCOR* | *DNMT3A* | *IKZF1* | *NRAS* | *SRSF2* |
| *BCORL1* | *ETV6/TEL* | *JAK2* | *PDGFRA* | *STAG2* |
| *BRAF* | *EZH2* | *JAK3* | *PHF6* | *TET2* |
| *CALR* | *FBXW7* | *KDM6A* | *PTEN* | *TP53* |
| *CBL* | *FLT3* | *KIT* | *PTPN11* | *U2AF1* |
| *CBLB* | *GATA1* | *KRAS* | *RAD21* | *WT1* |
| *CBLC* | *GATA2* | *MLL* | *RUNX1* | *ZRSR2* |
| *CDKN2A* | *GNAS* | *MPL* | *SETBP1* |  |

**Supplementary Table 3**. Monoclonal antibodies and fluorochromes used in the diagnostic flow cytometry panels

| Acute primary tube | CD34-ECD/CD3-FITC/CD7-PE/CD33-PC5.5/CD117-APC/Anti-HLA-DR-PB/CD10-PC7/CD19-APC-A750/CD20-APC-A700/CD45-KRO |
| --- | --- |
| Acute myeloid tube | CD34-ECD/CD11b-FITC/CD18-PE/CD13-PC5.5/CD33-PC7/CD117-APC/CD56-APC-A700/CD2-APC-A750/CD15-PB/CD45-KRO |
| Acute B tube | CD45-KRO/CD19-APC-A750/CD22-PB/CD38-APC-A700/CD10-PC7/CD79b-APC/CD13-PC5.5/CD34-ECD/Anti-Kappa-FITC/Anti-Lambda-PE |
| Acute T tube | CD1a-FITC /CD7-PE/CD34-ECD/CD13-PC5.5/CD16-PC7/CD4-APC/CD5-APC-A700/CD2-APC-A750/CD8-PB/CD45-KRO |
| Acute intracellular tube | CD34-PC7/Anti-TDT-FITC/Anit-MPO-PE/CD79a-APC/CD3-PB/CD45-KRO |
